# Assessment, patient selection, and rehabilitation of nerve transfers

**DOI:** 10.3389/fresc.2023.1267433

**Published:** 2023-11-20

**Authors:** Emma A. Bateman, Juliana Larocerie-Salgado, Douglas C. Ross, Thomas A. Miller, Stahs Pripotnev

**Affiliations:** ^1^ Department of Physical Medicine & Rehabilitation, Schulich School of Medicine & Dentistry, Western University, London, ON, Canada; ^2^ Parkwood Institute, St Joseph’s Health Care London, London, ON, Canada; ^3^ Roth McFarlane Hand & Upper Limb Centre, St Joseph’s Health Care London, London, ON, Canada; ^4^ Division of Plastic & Reconstructive Surgery, Department of Surgery, Schulich School of Medicine & Dentistry, Western University, London, ON, Canada

**Keywords:** nerve transfer, nerve transfer rehabilitation, electromyography, peripheral nerve injury, rehabilitation

## Abstract

Peripheral nerve injuries are common and can have a devastating effect on physical, psychological, and socioeconomic wellbeing. Peripheral nerve transfers have become the standard of care for many types of peripheral nerve injury due to their superior outcomes relative to conventional techniques. As the indications for, and use of, nerve transfers expand, the importance of pre-operative assessment and post-operative optimization increases. There are two principal advantages of nerve transfers: (1) their ability to shorten the time to reinnervation of muscles undergoing denervation because of peripheral nerve injury; and (2) their specificity in ensuring proximal motor and sensory axons are directed towards appropriate motor and sensory targets. Compared to conventional nerve grafting, nerve transfers offer opportunities to reinnervate muscles affected by cervical spinal cord injury and to augment natural reinnervation potential for very proximal injuries. This article provides a narrative review of the current scientific knowledge and clinical understanding of nerve transfers including peripheral nerve injury assessment and pre- and post-operative electrodiagnostic testing, adjuvant therapies, and post-operative rehabilitation for optimizing nerve transfer outcomes.

## Introduction

Nerve injuries are common and can have devastating effects on an individual's physical, psychological, and socioeconomic wellbeing (1–4). The management of these injuries has come a long way since the days of Hippocrates and Galen when nerves were thought to be irreparable (5, 6). It wasn't until the 18th century that nerves were proven to regenerate (7, 8), and not until the first and second world war that more attention was brought to nerve repair (9, 10). While microsurgical technology advanced, nerve repair outcomes improved and nerve grafts became standard practice for reconstructing gaps (11). As indications expanded, it became clear that even these techniques have limitations, namely worse outcomes in proximal injuries and injuries with larger nerve gaps, due in part to the relatively fixed rate of axonal regrowth and the finite window for reinnervation of the target organ muscle. In addition, grafting of longer nerve gaps increases the likelihood of mismatch between motor and sensory axons. Consequently, nerve transfers were introduced.

Nerve transfers involve coapting a healthy expendable donor nerve to the end or side of the injured nerve. This can be done outside the zone of injury and closer to the target end organ to restore the lost sensation or motor function (12, 13). Recent literature shows superior outcomes in patients treated with nerve transfers compared to conventional techniques, and they have become the gold standard treatment for certain injury patterns (14–16). This paradigm shift has brought us into the era of nerve transfers.

As our knowledge and understanding of nerve anatomy and regeneration expands, nerve transfer techniques continue to be developed and refined. Alongside this rapid growth in the field of nerve transfers has come the expansion of electrodiagnostic studies for diagnosis and management of these injuries (17–20). The management of nerve injuries does not start or end with the diagnosis or the surgery, but rather begins with preoperative optimization and continues into postoperative rehabilitation, both of which can have a significant impact on patient outcomes (21, 22). This narrative review will discuss (1) clinical evaluation of peripheral nerve injury; (2) electrodiagnostic evaluation of peripheral nerve injury; (3) indications for and timing of nerve transfer surgery; (4) adjuvant treatments; (5) post-operative rehabilitation; (6) post-operative electrodiagnostic evaluation; and (7) timelines for recovery.

## Peripheral nerve injury clinical evaluation

The assessment of nerve injuries begins with a thorough history and physical examination which can often be enough to diagnose specific deficits. Prognostically, determining the timing and etiology of the nerve injury is critical. Nerve injuries can be broadly classified into open (i.e., sharp, penetrating) and closed (i.e., blunt trauma, stretch, avulsion, crush) injuries. Injury mechanism plays an important role in understanding the potential severity and predicted recovery. For instance, sharp penetrating injuries confer a higher suspicion for complete nerve transection and are typically explored and repaired early. Conversely, gunshot penetrating and closed injury mechanisms are typically followed clinically to monitor for spontaneous improvement and to further characterize the degree and distribution of the injury.

Physical examination of motor and sensory deficits characterizes the sequelae of nerve injury. Sensation can be assessed with a variety of techniques including light/sharp touch, the ten test, two-point discrimination, and Semmes Weinstein monofilaments, all of which have advantages and limitations (23–27). Identifying abnormal sensation is particularly important to complement the electrodiagnostic assessment, described below, as sensory loss in areas of normal sensory electrodiagnostic testing suggests a pre-ganglionic injury (28). Motor assessment is done with a focused examination of each muscle group in the upper and/or lower extremity, and is most commonly reported based on the Medical Research Council (MRC) scale (29). Attention is paid to sensory and motor deficits based on both peripheral nerve distribution as well as dermatomal/myotomal distribution depending on the injury pattern. Provocative testing with focal pressure or the Hoffman-Tinel sign along the course of a nerve can be used to identify the location of an injury, to follow recovery, or to reveal secondary points of compression at known entrapment sites (30–33). Combined with serial clinical assessments, electrodiagnostic testing provides insight into the diagnosis and localization, severity, prognosis, and recovery progress to help assess spontaneous recovery and guide post-operative recovery and rehabilitation.

In some cases, imaging modalities offer further diagnostic and prognostic value to clinicians' pre-operative evaluation. Magnetic resonance (MR) and ultrasound imaging modalities are the most often utilized for their superior soft tissue resolution and have multiple applications in a patient with suspected nerve injury (34). Conventional MR images as well as MR neurography, which involves dedicated MR evaluation of peripheral nerves, can identify nerve root avulsions and focal abnormalities, respectively (35). Confirmation of the presence of preganglionic injuries using conventional MR of the cervical and/or lumbosacral spine, especially in patients with suspected concurrent pre- and post-ganglionic injuries, can further clarify prognosis and enhance surgical planning. Conventional MR and/or MR neurography can localize lesions within peripheral nerves at sites where clinical and/or electrodiagnostic evaluation will not, such identifying the precise site of injury in the median nerve proximal to the elbow (36). Like MR neurography, ultrasound can improve localization of peripheral nerve injuries by identifying more precise sites of injury when they are difficult to elucidate on clinical and/or electrodiagnostic assessment (34). Ultrasound has the additional advantage of allowing for real-time and dynamic evaluations, such as subluxation of the ulnar nerve at the elbow. As technology continues to drive advancements in medical imaging, these and novel techniques, such as electrosonomyography, may play a greater role in both pre- and post-operative assessments of nerve injury (37). However, not all patients need and/or benefit from imaging and clinicians should weigh carefully whether these investigations will change management (38, 39).

## Pre-operative electrodiagnostic assessment

Together with a thorough history and physical examination, electrodiagnostic testing (EDX) serves an important role in optimizing outcomes after nerve injury. Although there are indications for proceeding to surgery without EDX, such as in sharp/penetrating nerve injuries which warrant urgent/emergent exploration and repair, most patients undergoing nerve transfer procedures benefit from pre-operative planning that includes electrodiagnostic evaluation (40). Due to the pathophysiology of nerve injury, initial EDX is typically not useful earlier than 1.5 weeks post-injury and is most instructive at least 3–4 weeks post-injury (17).

Pre-operative EDX serves three main purposes: first, to characterize the extent of nerve injury including affected and unaffected nerves; second, to characterize the severity of the nerve injury and provide prognostic information to identify targets for nerve transfer; and third, to identify the availability of suitable donor nerves.

As a complement to the history and physical examination, EDX using nerve conduction studies (NCS) and needle electromyography (EMG) delineates which muscles and which sensory nerves are affected or spared. Because of normal anatomical variants in peripheral nerve anatomy, and because nerve injuries may spare some nerve fascicles, these electrodiagnostic techniques provide important diagnostic clarification as to the extent of nerve involvement and can be used to establish the approximate localization of injury, if not already known.

In addition to establishing the extent of nerve injury, EDX serves to characterize nerve injury severity. Using NCS and EMG after sufficient time has elapsed (minimum 7–10 days, optimally 3–4 weeks) can further characterize whether the injury pathology is demyelinating, axonal, mixed, or pre-ganglionic. For instance, in the setting of a suspected lower trunk brachial plexopathy, the presence of reduced motor NCS responses and denervation potentials with reduced or absent motor recruitment for the ulnar and thenar muscles together with normal sensory NCS responses at the dorsal ulnar cutaneous and medial antebrachial cutaneous nerves suggests a pre-ganglionic injury such as a C8/T1 nerve root avulsion or upper motor neuron injury (41). For post-ganglionic injuries, injury severity is typically defined using the Seddon and/or Sunderland classifications (Table 1).

**Table 1:** Classification of post-ganglionic nerve injury severity.

Seddon classification	Sunderland classification	Definition and key features	Recovery mechanism(s)	Prognosis
Neurapraxia	Grade 1	Demyelination without axonal loss	Remyelination	Full recovery; <3 months
Axonotmesis	Grade 2	Injury to axon without damage to supporting nerve architecture; Wallerian degeneration occurs	Axon sprouting; axonal regrowth; muscle fibre hypertrophy	Partial to full recovery (1 mm/day); prognosis favourable if motor/sensory NCS present and EMG recruitment is only mildly reduced
	Grade 3	Injury to axon and endoneurium causing intrafascicular disorganization; Wallerian degeneration occurs		
	Grade 4	Injury to myelin sheath, axon, endoneurium, and perineurium causing massive internal disorganization; Wallerian degeneration occurs	Axonal regrowth; muscle fibre hypertrophy	Poor; may not recover without surgery
Neurotmesis	Grade 5	Disruption of entire nerve architecture; transection; Wallerian degeneration occurs	No recovery without surgical intervention	

However, electrodiagnostic testing does have limitations, such as being unable to distinguish purely post-ganglionic injuries from mixed pre- and post-ganglionic injuries and being unable to distinguish axonotmetic from neurotmetic injuries in a single evaluation. Moreover, sensory NCS may overestimate the severity of post-ganglionic nerve injuries in mixed motor/sensory nerves (28). As a result, EDX alone is insufficient to guide next steps and is more instructive serially, and as a complement to a comprehensive history and physical exam (42).

By establishing the severity of injury, the electrodiagnostic evaluation confers prognostic information about likely recovery to guide management. Predominantly demyelinating (neurapraxic) nerve injuries typically recover within weeks to months; nerve transfers are therefore unlikely to offer functional improvements. Conversely, proximal axonal injuries (whether axonotmetic or neurotmetic) are unlikely to undergo spontaneous and complete recovery within 12–18 months if more than 15–20 cm (6–8 inches) from their target organ, as in brachial plexopathies (43). Consequently, these affected nerves could be candidates for nerve transfer to improve the likelihood of functional restoration. Often, the decision-making is more complex than in the aforementioned clinical scenarios. For instance, in cases of axonotmesis with axonal continuity to the target organ evidenced by 1–2 motor units on EMG or present but markedly reduced amplitude motor or sensory responses on NCS, the optimal management strategy may involve serial electrodiagnostic assessments every 1–3 months to monitor nerve recovery (44). This close follow-up permits monitoring of the trajectory of nerve recovery clinically and electrodiagnostically to increase confidence in the likelihood of satisfactory spontaneous recovery or the need for surgical intervention (45).

In addition to the insights about extent of injury, approximate localization of injury, and which nerves would benefit from transfer, EDX serves as an invaluable tool to identify suitable donor nerves. Nerve transfer requires a healthy, expendable donor nerve, which typically serves a redundant or less crucial function (45, 46). Needle EMG can confirm the health of the donor nerve and thus its suitability for transfer. For instance, a common nerve transfer for isolated posterior interosseus nerve (PIN) injuries is the supinator to PIN “supercharged end-to-side” (SETS) or end-to-end (ETE) nerve transfer (47). Depending on the etiology of nerve injury, a more proximal radial nerve injury affecting supinator may be difficult to exclude without needle EMG of the supinator muscle. The magnitude of recruitment on needle EMG testing, along with clinical evaluation of potential donor nerves' muscle power, may be useful adjuncts in decision-making since donor nerves that are functioning but injured may produce less satisfactory results (48). Moreover, to avoid surgery resulting in donor morbidity (in this case, weakness of forearm supination), needle EMG evaluation of the biceps brachii can provide reassurance that the patient will not lose supination function with the supinator nerve transfer. Thus, in cases where nerve transfer is being considered, electrodiagnostic evaluation of the potential donor nerve(s) and nerve(s) performing the same function as the putative donor nerve(s) is critical to ensure suitable donor selection and avoid functional loss. Electrodiagnostic evaluation can also guide the need for and timing of surgical intervention, use of adjuvant therapies, and rehabilitation to optimize outcomes from nerve injury and/or nerve transfers.

## Indications and timing for nerve transfer surgery

Indications for nerve transfers continue to expand and evolve but in general include nerve root avulsions, proximal injuries where the distance to reinnervation is expected to be too long for meaningful recovery, multi-level injuries, large nerve gaps, and situations where it is not safe or feasible to operate in the original zone of injury (12, 13). Garg et al. and Yang et al. both demonstrated in systematic reviews that nerve transfer is superior to traditional root nerve grafting in upper brachial plexus injuries (14, 16). However, other similar systematic reviews comparing nerve transfer to traditional reconstructive techniques in obturator, tibial, axillary, and radial nerve injuries found traditional reconstructive techniques and nerve transfers had comparable results (49–53).

Another indication for nerve transfers is in upper limb reconstruction post-spinal cord injury (SCI) as stand-alone procedures or in conjunction with tendon transfers (18, 54). In contrast to other nerve transfer indications, both the donor and recipient peripheral nerves are typically intact in persons with SCI and the nerve dysfunction stems from upper motor neuron injury; the purpose of the nerve transfer is to reinnervate paretic muscle using a dispensable donor peripheral nerve whose myotome is above the neurological level of SCI. For instance, a supinator nerve branch can be transferred to the posterior interosseous nerve (PIN) to restore finger extension in an individual with C6 level SCI because the supinator receives is myotomal innervation from C5/6 (above the level of SCI), whereas the finger extensors are primarily innervated by C7/8 (54).

Indications for specific nerve transfer techniques are also evolving. SETS transfers remain somewhat controversial. The goal with SETS transfers is to supplement existing nerve function with new axons, “babysit” distal motor endplates, reduce Schwann cell degeneration, and provide additional growth factors, all while avoiding the sacrifice of possible proximal nerve recovery as is necessary with standard ETE transfers (21, 55–61). A randomized controlled trial by Xie et al. compared 45 patients treated with anterior interosseus nerve (AIN) to ulnar motor SETS transfer in addition to a subfascial ulnar transposition at the elbow with 48 patients who only received the transposition (62). At a final 2 year follow up, they found that patients in the SETS treatment group had significantly better pinch strength and compound motor action potentials (CMAP) on electrodiagnostic studies (62). A systematic review of 16 studies by Thakkar et al. also supported improved outcomes in SETS AIN to distal ulnar nerve transfers compared to traditional techniques (15).

For any nerve injury, the timing of surgical intervention is critical: denervated muscles undergo atrophy and after a certain amount of time, motor end plate loss is irreversible and reinnervation does not occur (63). Ideally, reinnervation after peripheral nerve injury is achieved within 12–18 months of the injury. Although early reports of nerve transfer in SCI opined that the intact neural loop below the neurological level of injury protects against denervation, thereby prolonging the potential period for nerve reconstruction, it has been increasingly recognized that many patients with SCI have concomitant lower motor neuron injuries around the neurological level of injury (also termed the level of SCI or lesional level) (19, 64). In these patients, the same urgency for reconstruction exists for restoring innervation to muscles whose myotomal innervation is within this lesional area as it does for patients with typical post-ganglionic injuries (19, 64). Electrodiagnostic studies are a powerful tool to help guide decision making, including for the timing and/or need for intervention.

## Adjuvant therapies in nerve transfer surgery

This section outlines the role of adjuvant therapies, defined as interventions which occur outside the operating room and are designed to improve outcomes in nerve reconstruction. Although they may be considered adjuvant therapies, this portion of the review will not include formal therapy before (“pre-hab”) or after surgery as these topics are covered elsewhere in this paper. This portion of the review also does not review locally applied (i.e., intraoperatively) substances that have been shown to enhance outcomes in the laboratory but for which efficacy in humans is lacking.

The factors which lead to suboptimal outcomes after nerve injury and repair have been elucidated in a series of elegant studies (65, 66). A number of possible adjuvant therapies putatively targeting these factors have been observed to be efficacious in the laboratory setting but evidence for improvement in humans is lacking. Examples include atorvastatin and L-carnitine (67). Both have been shown to have neuroprotective effects in CNS injury and degenerative models in rat models. Both have also been shown to improve outcomes in a rodent sciatic nerve injury model, but human trials and data are not available. Similarly, Gordon and Borschel demonstrated that locally delivered glial derived neurotrophic factor (GDNF) enhanced axonal regeneration, axonal maturation and final muscle contractility after sciatic nerve repair in a rat model (68). However, human trials are not available or pending.

Adjuvant therapies trialed in human subjects include platelet-rich plasma (PRP), tacrolimus (FK506), and electrical stimulation. PRP has gained widespread use in the treatment of various musculoskeletal conditions such as lateral epicondylitis and Achilles tendon rupture (69). PRP contains significant concentrations of growth factors which have been demonstrated to promote Schwann cell proliferation and migration; thus, there is some theoretical basis for its use after nerve repair. However, currently there are only very small studies examining the outcomes in nerve compression—specifically carpal tunnel syndrome. While outcomes did appear to be enhanced, treatment numbers were small (70). At this time, there is no evidence that adjuvant treatment with PRP enhances recovery after nerve transfer or repair.

FK506 (tacrolimus) is a potent inhibitor of T-cell mediated immunity and is widely used in solid organ transplantation as an immunosuppressant. When given systemically, FK506 appears to enhance the rate of nerve regeneration and may act via other mechanisms such as diminishing neural scarring. Because of the potential toxicities of FK506, combined with a narrow therapeutic dosage window, some investigators have worked on delivery of the drug at the site of nerve injury via delivery systems such as microspheres and nerve conduits (71, 72). Results have been encouraging, albeit from few small clinical trials, but currently there is no commercially available system with regulatory approval.

Of all adjuvant therapies, brief electrical stimulation (ES) has shown the most promise and is closest to widespread clinical availability. Electrical stimulation enhances the rate of axonal growth after nerve injury. A number of methods of applying electrical stimulation have been described in both animal models and humans (73, 74). In clinical practice, groups have examined various durations of electrical stimulation immediately post-surgery. Stimulation times varying from 10 min to 1 h appear to have similar efficacies (73). Improvements in outcomes have been demonstrated in various nerve injury models including carpal tunnel syndrome (75), cubital tunnel syndrome (76), and digital nerve lacerations (77).

More recently, even greater enhancements in nerve recovery have been demonstrated with pre-operative (“pre- conditioning”) ES. Clinically, this is likely to be more practical and cost-effective than applying ES peri-operatively. Senger et al. have demonstrated improvements in both a nerve graft and nerve transfer rat model which appears to be superior to post-operative ES (78, 79). While ES appears to be efficacious, widespread adoption awaits larger clinical trials and more easily used, commercially available devices to deliver the stimulation.

In summary, various systemically given or locally applied pharmacologic substances hold promise to enhance recovery after nerve injury and repair. However, currently, none are available for routine, practical use. Brief electrical stimulation shows real promise as an adjuvant therapy. Larger clinical trials and routine use await easily available and utilized devices. All adjuvant treatments require more robust scientific evidence to determine which are truly effective and how they can be incorporated into nerve reconstruction techniques, including nerve transfers, to optimize patient outcomes.

## Rehabilitation post-nerve transfer

After nerve transfer, rehabilitation plays a crucial role in optimizing recovery and patient outcomes. Although the focus of this review is on addressing motor dysfunction with nerve transfers, addressing sensory deficits is important for patients' outcomes. Patients with sensory deficits often benefit from sensory relearning programs, a field with established techniques (80). Clinicians should consider these techniques when sensory function is expected to improve spontaneously or post-surgically. A detailed review of these techniques falls outside the scope of this review. Similarly, a comprehensive dissection of differences, advantages, and disadvantages of nerve transfer compared to conventional nerve grafting falls outside the scope of this review; however, readers should be aware that nerve transfers for restoration of motor function often do not offer potential for sensory reinnervation. Regardless of the expected trajectory or prognosis for sensory recovery, clinicians should counsel patients with sensory deficits on protective and compensatory strategies for insensate areas while encouraging the use of the hand in activities of daily living as well as vocational and avocational activities unless there is a contraindication (80). Education about cold sensitivity and strategies such as glove or mitten use outdoors, particularly for patients in colder climates, may also be of benefit for patients with sensory impairment. In addition, for patients experiencing hypersensitivity and allodynia, clinicians should consider using established non-pharmacologic and pharmacologic strategies (80–82). Motor rehabilitation following nerve transfers consists of five phases based on stages of nerve healing, cortical reorganization, strength, and endurance, as outlined below and in Table 2 (22, 80–82).

**Table 2:** Summary of motor rehabilitation phases post-nerve transfer.

	Phase	Timeframe	Objectives
Pre-reinnervation	1) Protective	0–3 weeks post-op	• Protect nerve coaptation • Control edema
	2) Corrective (silent)	3 weeks post-op (MRC 0) to MRC 1	• Address residual deformities • Maintain passive ROM • Stimulate function of remaining muscles • Stimulate cortical awareness of donor-recipient correlation
Post-reinnervation	3) Re-education	MRC 1–2	• Establish donor-recipient nerve co-activation • Start active ROM in gravity-eliminated positions • Stimulate functional use of new motion
	4) Strengthening	MRC 2–3	• Perform active ROM against gravity • Increase functional use of new motion • Decrease reliance on donor nerve (donor de-activation) • Facilitate selective activation of donor and recipient muscles
	5) Endurance (function)	MRC >3	• Perform active ROM against light/moderate resistance • Increase resistance and endurance • Facilitate return to pre-injury function/work

MRC, medical research council; ROM, range of motion.

The goal of nerve transfer rehabilitation is to restore function and independence through both cortical and peripheral plasticity to perform daily activities that were once lost.

### Phase 1: protective phase

In the protective phase, during the first 3 weeks following nerve transfers, avoiding excessive tension on the neurorrhaphy through active or passive motion is the crucial consideration. Although the recipient and donor nerves are loosely coapted, orthotic intervention may be necessary to ensure minimal tension on the neurorrhaphy (83, 84). In addition to protecting the nerve coaptation, patients are instructed in retrograde massage, compression stockings, positioning to control edema, and careful range of motion (ROM) exercises to maintain mobility (21, 85).

Orthoses that patients may have worn before surgery for positioning and/or increasing ROM can be reintroduced in this phase, provided they do not impose tension on the nerve coaptation.

### Phase 2: silent/corrective phase

**Figure 1:**
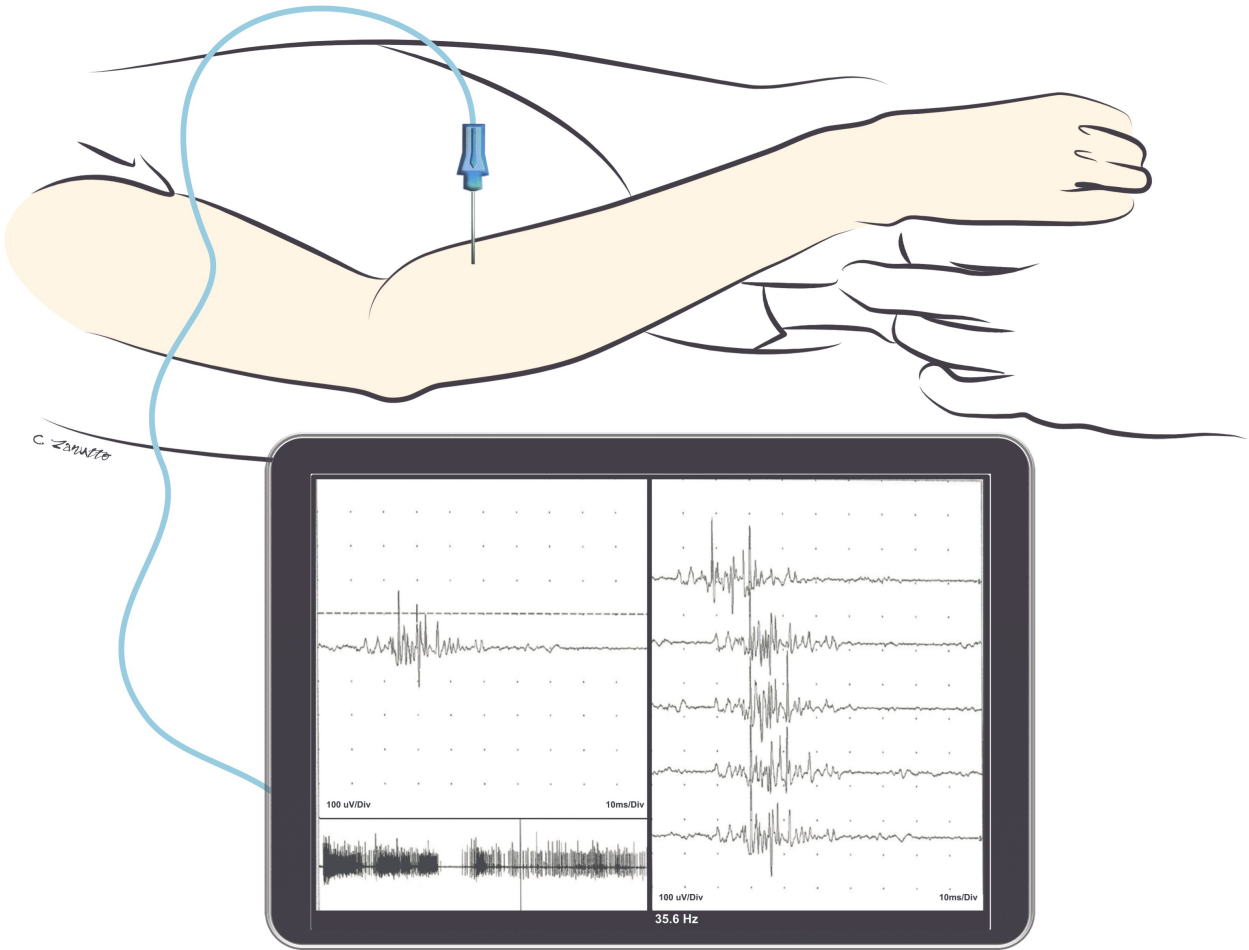
Triggered nascent motor unit action potential seen during needle EMG in the extensor digitorum communis muscle 10 weeks after nerve transfer (median nerve fascicle to flexor digitorum superficialis to the posterior interosseous nerve). Note that the nascent unit is appreciable with finger flexion as the patient activates the donor nerve. Nascent motor unit action potentials are characterized by their small amplitude, long duration, polyphasia, and instability.

The period between 3 weeks post-operative and the beginning of early reinnervation is known as the “silent phase” due to the lack of active motion (86, 87). Early reinnervation is indicated by the presence of polyphasic nascent (newborn) motor unit potentials (Figure 1) on electromyography and clinically palpable muscle contractions (MRC grade 1). At our centre, the “silent” period typical of this phase is an opportunity to strengthen functioning muscles, and address contractures, deformities, and tightness, hence adding a “corrective” component to this phase in the recovery process. Patients will be guided in passive ROM exercises, as well as the use of static and static progressive orthoses to correct or minimize issues associated with minimal or absent muscle function (22).

Although no active motion is observed during this period, cortical reorganization (neuroplasticity) may be stimulated through motor imagery (MI), as new connections need to be established for good motor function of recipient muscles (21, 85, 86, 88, 89). MI is the imagined representation of a movement without its physical execution. It activates neurons in brain structures involved in physical movements, such as the primary motor cortex, cerebellum, and basal ganglia (87, 88, 90). After nerve transfers, MI involves mentally visualizing both donor and recipient actions combined in one motion, which Kahn and Moore (2016) refer to as “donor flooding exercises” during the silent/corrective phase (21).

### Phase 3: retraining phase

Retraining begins with early reinnervation (MRC 1) until the patient demonstrates active range of motion in a gravity-eliminated position (MRC 2). This phase is characterized by a greater number of immature polyphasic motor units on needle EMG. The primary therapeutic goal is to facilitate motor relearning and cortical reorganization (cortical plasticity) to help patients reproduce the newly targeted motion independently. This is initially done by so-called “donor activation” in which the patient activates the original target muscle of the donor nerve to produce motion in the recipient muscle. For instance, in the case of the flexor digitorum superficialis fascicle of the median nerve used as a donor to reinnervate brachialis, elbow flexion may be initiated by concomitant finger flexion (22, 91, 92).

To assist in this process, EMG-biofeedback is a valuable modality that provides patients with visual and auditory information about targeted recipient muscle activity, even before any clinically appreciable muscle function is noticed (93). When combined with donor activation exercises, EMG-biofeedback can effectively assist with motor relearning and enable patients to elicit newly acquired motion. Initially, EMG-biofeedback is introduced to reach 50% of the maximum volitional contraction and gradually increased as the patient progresses (22, 86, 89).

Mirror therapy (MT) is also valuable for stimulating cortical plasticity. MT was initially described by Ramachandran for treating phantom limb pain in amputees (94, 95). It has since been used for various conditions to facilitate motor function by activating the contralateral motor cortex. Observing the reflection of the unaffected hand moving, the cerebral areas relevant for somatosensory processing are stimulated, integrating visual, somatosensory, and motor networks (88, 96, 97). Clinical trials have shown that MT can effectively manage pain and paraesthesias following open carpal tunnel release (98). In addition, a study comparing the effects of mirror therapy vs. classic sensory re-education on sensorimotor recovery and cortical activation after nerve injuries on the forearm demonstrated greater bilateral associations and cortical activation in the MT group (90). Therefore, the mirror illusion of the moving limb combining donor and recipient functions facilitates cortical reorganization and motor relearning.

**Figure 2:**
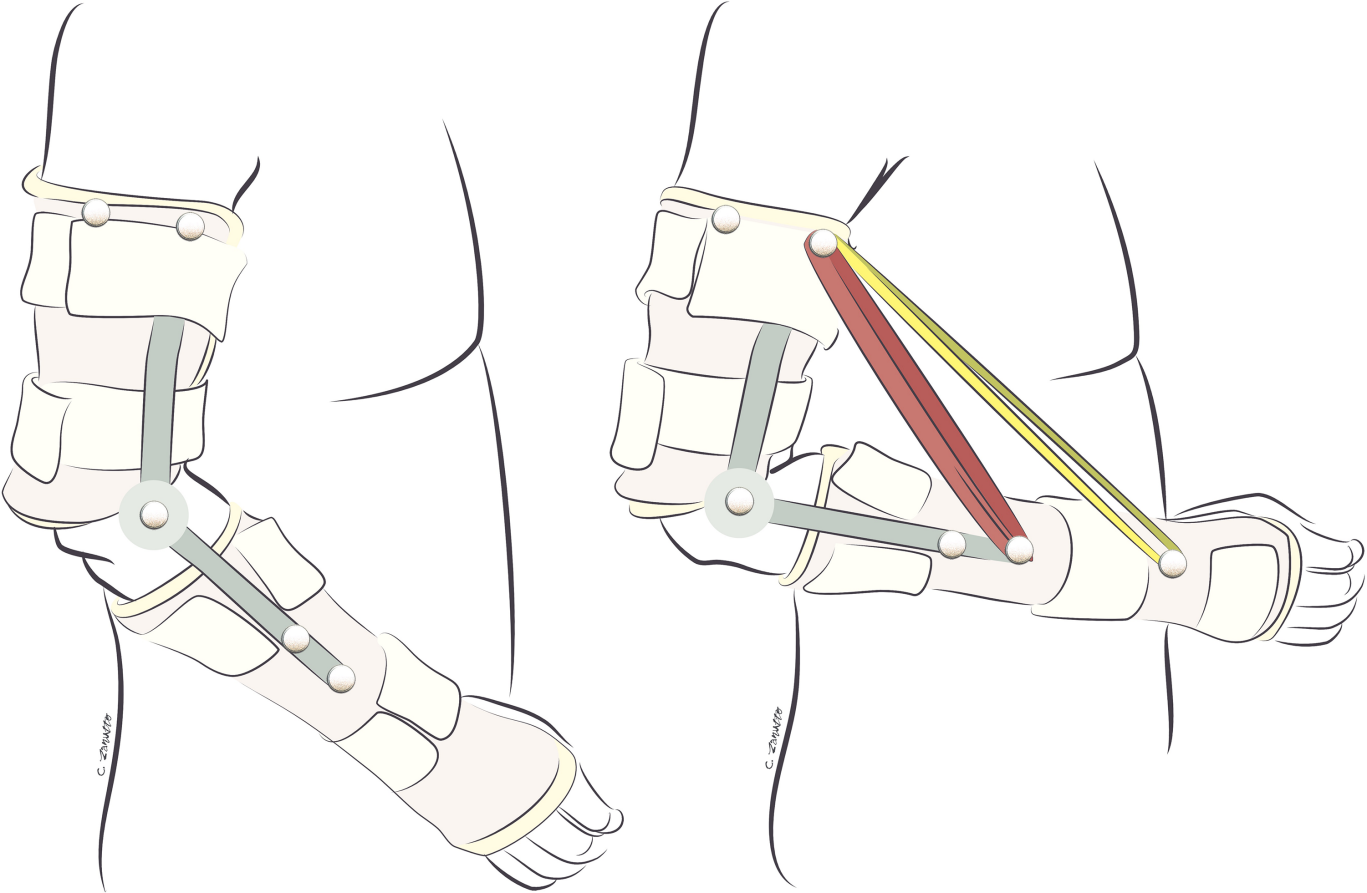
Dynamic-assist orthosis to augment elbow flexion after double fascicular nerve transfer to restore elbow flexion. On the left, the patient is maximally contracting the elbow flexors but cannot move the arm against gravity. On the right, the assistance of rubber bands on the dynamic-assist orthosis allows the paitent to flex the elbow against gravity.

**Figure 3:**
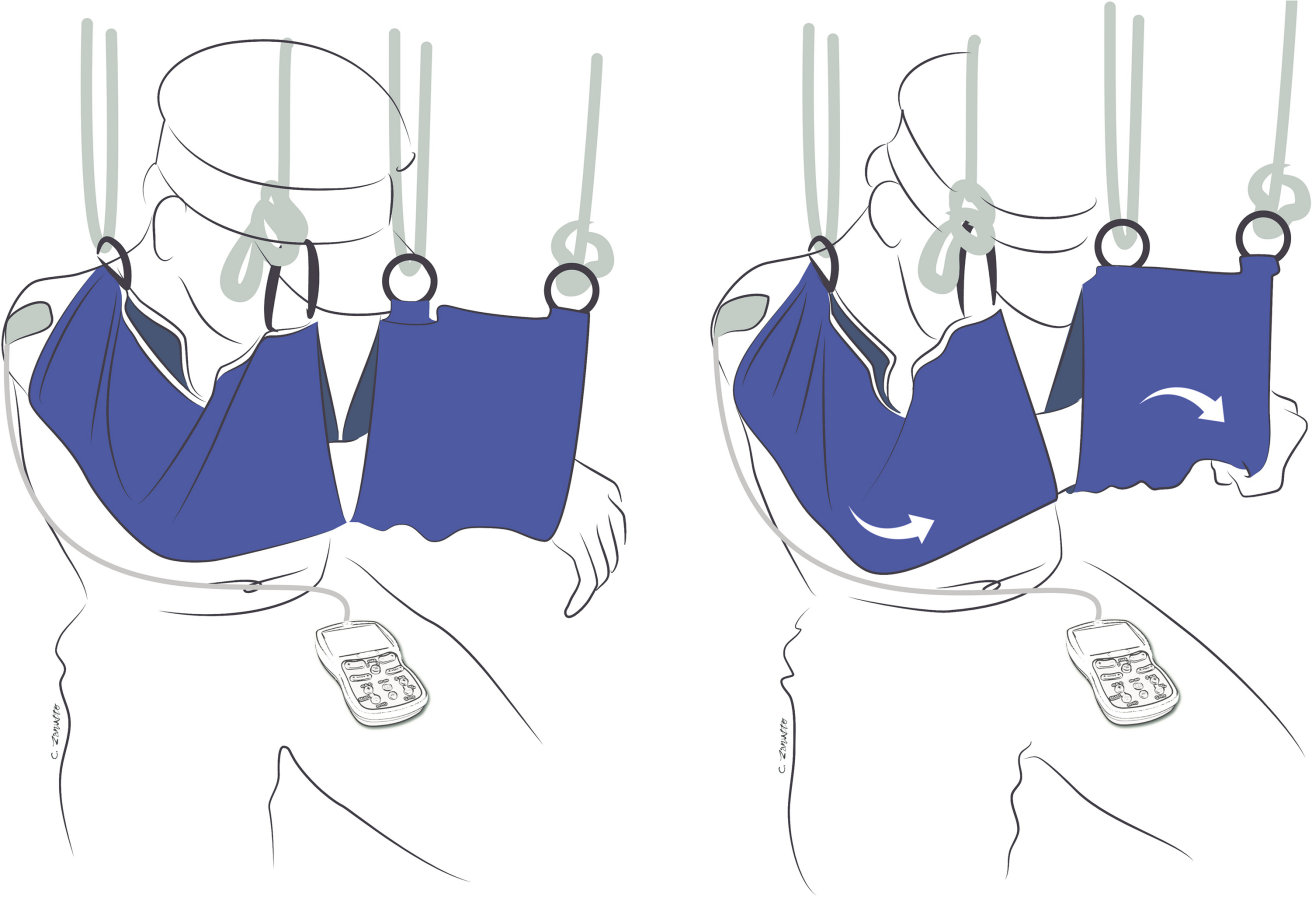
Gravity-eliminated exercises combining donor and recipient muscle function to flex the elbow with the assistance of suspension slings to eliminate gravity and EMG-biofeedback to improve nerve activation after double fascicular transfer (ulnar nerve fascicle to flexor carpi ulnaris to musculocutaneous nerve branch to brachialis and median nerve fascicle to flexor digitorum superficialis to musculocutaneous nerve branch to biceps). On the left is the patient's starting position. On the right, the patient is flexing the elbow while concurrently flexing the wrist and fingers to activate the donor nerves.

Exercises in this phase are performed in gravity-eliminated or gravity-lessened positions, with or without the assistance of dynamic-assist orthoses or sling/suspension systems, as illustrated in Figures 2, 3, respectively (21, 22). Dynamic-assist orthoses can reduce the effect of gravity on recovering weak muscles and help with daily function, assisting weak muscles in moving a joint to a greater extent than their actual strength allows. The frequent activation of recovering muscles helps increase strength and endurance while concomitantly increasing cortical activation of the newly acquired motion (99). In some cases, such as double fascicular transfers to restore elbow flexion, patients may initiate this phase using suspension slings and gradually transition to primarily using a dynamic-assist elbow flexion splint like the one illustrated in Figure 2 (22).

### Phase 4: strengthening phase

Patients transition to the strengthening phase when their strength progresses from MRC grade 2–3, at which point patients can overcome gravity and some light resistance. This phase is characterized by a few mature and increasing numbers of polyphasic motor units on needle EMG studies. The main goal of this phase is to increase the strength and endurance of recipient muscles while consolidating the motor control of the newly acquired function (22).

In this phase, EMG-triggered electrical stimulation (EMG-ES) can be a highly effective tool. EMG-ES combines EMG-biofeedback with traditional neuromuscular electrical stimulation (NMES), requiring active patient participation to activate the neuromuscular stimulation (100). Hence, EMG-ES not only helps to increase muscle resistance but also facilitates the association between the donor nerve and recipient muscles' actions. This approach is superior to those where the onset of muscle activity is solely driven by a device, such as in NMES. In a study by Sardaru and colleagues, individuals with sciatica-related foot drop who underwent EMG-ES had slightly better results in muscle function, strength, and perceived disability than those who underwent NMES alone (101).

In denervated muscles, significant wasting and loss of motor units occur, leading to fatigue and potential adverse effects when newly innervated muscle fibers are overpowered. Therefore, lower frequency (i.e., 20 Hz) parameters are used to stimulate muscle fibers slightly above the threshold to produce tetanic contraction and below the threshold to stimulate anaerobic fibers. While the ideal functional electrical stimulation parameters in human models have not yet been determined, recent research has shown that peri-operative nerve stimulation at 20 Hz can improve recovery after nerve injury and repair (102–108).

**Figure 4:**
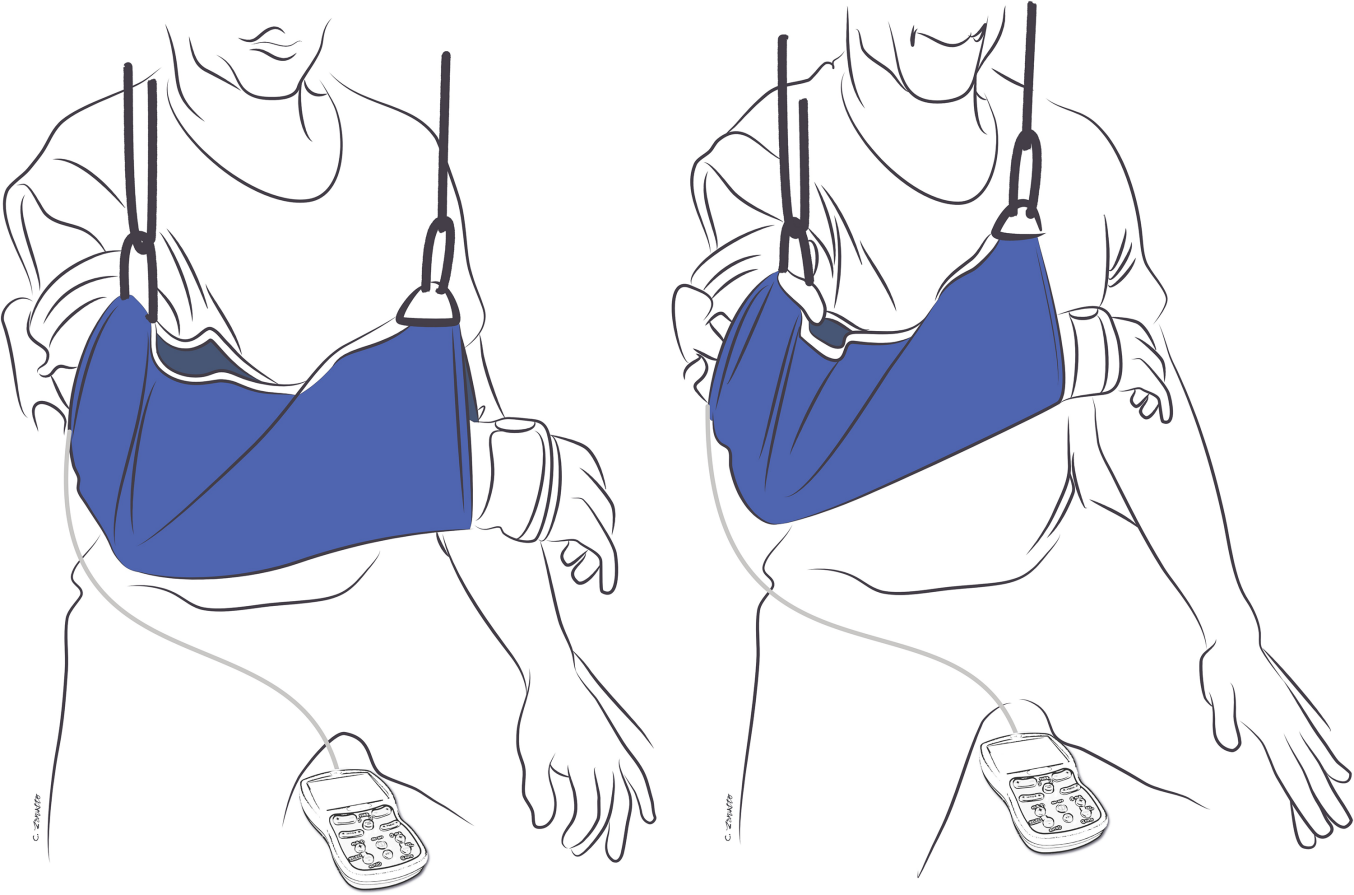
Selective activation or “donor de-activation” exercises. EMG-biofeedback of recipient muscle(s) occurs while simultaneously eliciting the recipient muscle(s) and the antagonist muscle(s) to the donor nerve. In this example, the patient has undergone double-fascicular transfer. On the left is the starting position with fingers relaxed bilaterally and the EMG-biofeedback of biceps below threshold. On the right is the final position, with bilateral finger and wrist extension while activating elbow flexors with the EMG-biofeedback of biceps above threshold.

As the quality of motion improves and the connection between donor and recipient nerves becomes established, the reliance on donor nerve activation to initiate new motion is expected to decrease. This, in turn, will increase the ability to selectively activate donor and recipient nerves separately (85, 87, 99). To achieve this, “donor de-activation” exercises are introduced during this phase to decrease the need for eliciting donor nerve function to initiate motion (22). By the end of this phase, patients should have undergone sufficient cortical reorganization to perform the action of the recipient muscle(s) without activating the donor nerve, and vice versa. For instance, in a double fascicular transfer in which fascicles of the median nerve to flexor digitorum superficialis are used to restore elbow flexion, donor deactivation is critical so patients can flex the elbow while keeping the digits extended, such as when washing their face, as well as flex the digits while keeping the elbow extended, such as when placing an object on a shelf (Figure 4).

**Figure 5:**
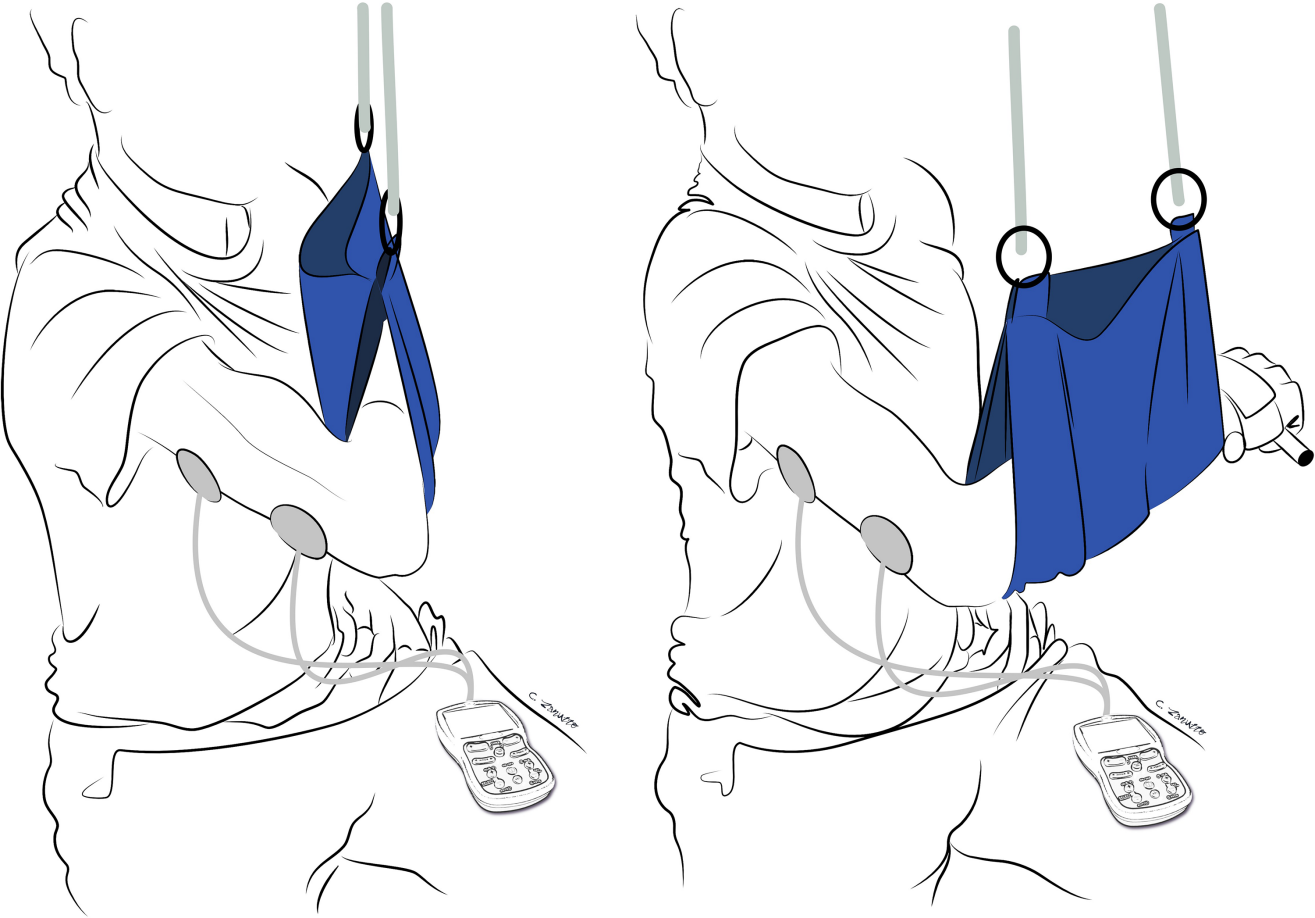
Selective activation or “donor de-activation” exercises. EMG-biofeedback of the antagonist muscle(s) to the recipient muscle(s) while simultaneously eliciting the function of the donor nerve and the antagonist muscle(s) to recipient muscle(s). In this example, the patient has undergone double-fascicular transfer. On the left is the starting position, fingers flexed holding an object and elbow flexed with EMG biofeedback on the triceps below threshold. On the right is the final position, with the patient demonstrating finger flexion (holding an object) while extending the elbow.

When performing donor de-activation exercises, EMG-biofeedback continues to be crucial in promoting cortical plasticity by providing feedback on muscle function. These exercises involve various techniques, including: (1) EMG-biofeedback of recipient muscle(s) while simultaneously eliciting contraction of the recipient muscle(s) and the antagonist muscle(s) to the donor nerve (Figure 4); (2) EMG-biofeedback of the antagonist muscle(s) to the recipient muscle(s) while simultaneously eliciting the function of the donor nerve and the antagonist muscle(s) to the recipient muscle(s) (Figure 5). By using these techniques, patients can better understand and control the activation of the donor and recipient muscles separately, without the need for donor nerve activation. This is a fundamental step in restoring natural and functional movement patterns (22).

**Figure 6:**
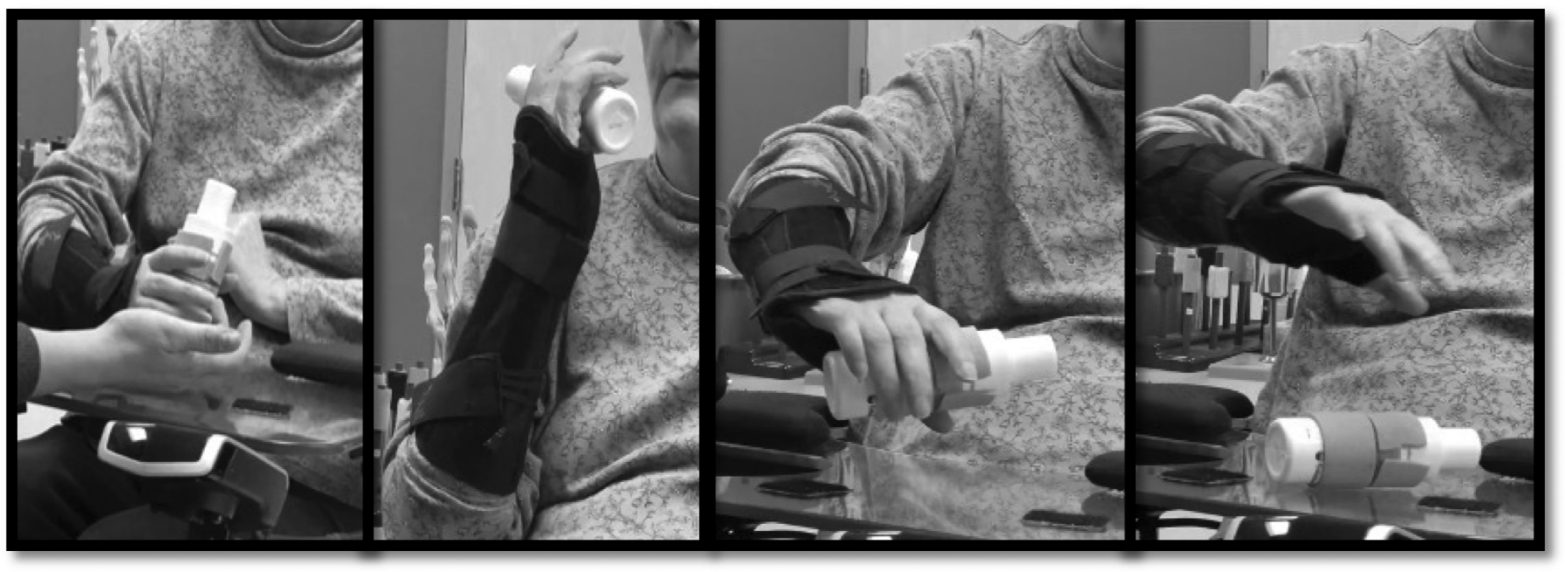
Selective activation or “donor de-activation” functional exercises in the late stages of the rehabilitation (endurance phase). In these photos, a person with SCI, who underwent supinator branch to posterior interosseous nerve transfer concurrent with musculocutaneous nerve branch to brachialis to anterior interosseous nerve fascicle, completes donor de-activation exercises. In the left two images, the patient is tasked with holding the object with fingers flexed while bringing the object to the mouth with elbow flexion and forearm supination. In the right two images, the patient is tasked with bringing the object back to the table and drop it by extending the fingers with the forearm in pronation. The patient is wearing a wrist orthosis to minimize wrist tenodesis while performing exercises.

It is essential to evaluate the muscles that antagonize the recipient muscles during nerve transfer rehabilitation, as weak muscles may be easily overpowered by the newly innervated muscles (Figure 6). For example, in patients with upper brachial plexus injuries who undergo nerve transfers to restore elbow flexion and primary nerve grafts for elbow extension, there may be a discrepancy in the degree to which these functional movements return and the timing at which this occurs. For instance, due to the distance between the injury site (supraclavicular brachial plexus) and triceps motor endplates, restoration of elbow extension may take longer than restoration of elbow flexion after double fascicular transfer. Moreover, there may be a discrepancy in the power restored for these functions, either temporarily or after maximal recovery, such as triceps regaining MRC 2–3 strength and elbow flexion regaining MRC 4 strength. As a result, clinicians may observe these patients flexing the elbow when reaching to grasp objects at arm's length. To address this, rehabilitation should also focus on strengthening the weak antagonist muscles while working on donor de-activation and selective activation strategies (22).

As muscle function and motor control improve during rehabilitation, reliance on dynamic-assist orthoses and support systems, such as suspension slings, gradually declines. However, compensatory movements may persist during exercises and functional use of the limb. To address this issue, orthoses and/or performing exercises in front of a mirror for visual feedback may be excellent adjuncts to reduce compensatory movements (85, 86, 90).

### Phase 5: endurance phase

This rehabilitation phase typically starts when patients can demonstrate full range of motion against gravity (MRC 3 and beyond) and can easily activate both donor and recipient nerves simultaneously. EMG studies indicate an increase in new and maturing motor units during this phase. The primary objective of this stage is to build endurance and facilitate the return to as many meaningful functional activities as possible.

NMES can be used as a standalone modality with similar parameters as the aforementioned EMG-ES to help increase resistance and endurance. MI is also implemented to help patients mentally replicate various tasks they need or desire to return to. During this phase, exercises are performed against gravity, without the assistance of dynamic orthoses, and with some resistance (109, 110).

It is worth noting that some patients may not have fully mastered the ability to activate donor and recipient nerves' function selectively. Therefore, in addition to building muscular strength and endurance, reducing reliance on donor activation for function should remain a critical therapeutic goal during this phase (22).

### Post-operative electrodiagnostic evaluation

Post-operative electrodiagnostic assessments fulfill two key functions after nerve transfer surgery. The first, primary function is to monitor for evidence of recovery; the second is to guide rehabilitation.

Post-operative electrodiagnostic assessments allow for monitoring of recovery following nerve transfer. Needle EMG is an effective tool to identify if motor nerve transfer has resulted in reinnervation of new axons to recipient muscles, evidenced by nascent units (Figure 1). Timing of these post-operative assessments intended to detect reinnervation requires careful consideration of the nerve transfer/reconstruction procedure and the expected time to reinnervation. Sufficient time for reinnervation correlates with the distance to reinnervation, as axonal regrowth occurs at a rate of approximately 1 mm per day.

The electrodiagnostic reassessment must focus on needle EMG of the most proximal muscle innervated by the recipient nerve, as this will be the first to be reinnervated. In addition, needle EMG must include attempts at activating the muscle through the donor nerve function (i.e., the function of the muscle whose nerve was transferred) and the recipient nerve function (i.e., the function that you have attempted to restore) (20, 111). For example, when assessing recovery of ulnar-innervated hand intrinsic muscles after AIN to ulnar motor SETS transfer, having the patient pronate the forearm (thereby firing the donor nerve to pronator quadratus) while the EMG needle is sampling the abductor digiti minimi or other ulnar-innervated hand intrinsic muscles is critical.

If sufficient time has passed and reinnervation should be evident on needle EMG and is not, then clinicians should consider re-assessment of surgical or other corrective options, such as tendon transfers or alternative nerve reconstruction procedures (111, 112). If insufficient time has passed, the absence of nascent units on needle EMG evaluation (or the absence of palpable or visible flicker of movement consistent with MRC 1 strength) may not be the result of failure of the nerve transfer surgery to achieve the intended outcome and later follow-up assessment is appropriate. Novel assessment methods, including combined use of ultrasound, stimulation, and needle EMG, are being investigated to improve diagnostic accuracy in these challenging situations (113).

In addition to monitoring for axonal regrowth, the post-operative electrodiagnostic assessment can guide rehabilitation. As outlined in the preceding rehabilitation section, the presence of multiple nascent units on needle EMG after the 3-week protective phase allows for progression from the Silent/Corrective Phase to the Re-education Phase. Beyond the re-education phase, needle EMG can also be used to determine to what extent a patient relies on donor activation. For instance, in the Silent/Corrective Phase, nascent units may only be detected with activation of the donor nerve function. Thereafter, there may be an appreciable difference in the number and recruitment of motor unit action potentials seen on needle EMG with greater motor unit activation and recruitment with donor and recipient co-activation than recipient nerve activation alone. As donor-recipient nerve co-activation improves and cortical reorganization occurs, this difference diminishes, and patients may have full interference patterns observable with recipient nerve activation alone. If this process is delayed or incomplete, and donor activation continues to be critical in the later phases of recovery, identifying this on needle EMG can help redirect therapy to address decreasing reliance on donor nerve activation.

The optimal frequency, number, and total duration of post-operative electrodiagnostic assessments is unknown. Those patients whose recovery would benefit from reassessment, whether through detection of reinnervation, confirmation of the need for alternative interventions, or to direct rehabilitation, may have multiple electrodiagnostic assessments in the post-operative periods at appropriate time intervals based on the expected trajectory of axonal regrowth.

### Timeline for recovery

As noted above, there is a finite window in which denervated muscle may undergo reinnervation. Consequently, particularly for very proximal nerve injuries, the decision to proceed with surgery must occur before the full extent of spontaneous recovery. This concept, as well as the expected timeline for recovery, require careful education for patients. The timeline for recovery after nerve injury, including for patients who undergo nerve transfer, can be lengthy. The aim of nerve transfer is to ensure reinnervation after peripheral nerve injury is achieved within 12–18 months of the injury. The initial time to reinnervation after nerve transfer is proportionate to the distance from the nerve transfer site to the muscle or sensory target, as nerves grow 1 mm per day. Therefore, in the immediate post-operative period, patients who have undergone nerve transfer will not see new motor or sensory functions. Once initial reinnervation is achieved, motor or sensory function may still be unsatisfactory until additional axonal growth and collateral sprouting have taken place. The time for patients to progress through the above phases of motor rehabilitation will vary based on patient factors, injury factors, the distance to reinnervation, and other factors. For some patients, recovery may still be incomplete despite surgical interventions to promote reinnervation.

## Conclusions

As the indications for, and use of, nerve transfers expand, the importance of pre-operative assessment and post-operative optimization intensifies. Comprehensive pre-operative history, physical examination, and, where appropriate, electrodiagnostic testing improve diagnosis and management of nerve injuries. Nerve transfer surgeries, whether alone or in conjunction with conventional nerve reconstruction/grafting or tendon transfer procedures, can improve functional recovery. Post-operative rehabilitation and electrodiagnostic assessments can optimize outcomes following nerve transfer. The future role of adjuvant treatments, including ES, have yet to be precisely elucidated but show promise for further enhancing recovery from peripheral nerve injury.
